# Off-target effects of protein tyrosine phosphatase inhibitors on oncostatin M-treated human epidermal keratinocytes: the phosphatase targeting STAT1 remains unknown

**DOI:** 10.7717/peerj.9504

**Published:** 2020-08-14

**Authors:** Brian V. Hong, Ji H. Lee, Robert H. Rice

**Affiliations:** Department of Environmental Toxicology and Forensic Science Program, University of California, Davis, CA, United States of America

**Keywords:** Cytokines, PVSN, STAT1, Transglutaminase, Vanadate

## Abstract

Cytokine signaling in the epidermis has an important role in maintaining barrier function and is perturbed in pathological conditions. Environmental exposures, such as to metal compounds, are of interest for their potential contribution to skin disease. Present work explores the possibility that vanadate is a more effective protein tyrosine phosphatase inhibitor in human keratinocytes than previously observed in fibroblasts. It focuses on the state of phosphorylation of signal transducer and activator of transcription 1 (STAT1) on tyrosine 701 upon treatment of cultured human keratinocytes with the cytokine oncostatin M, a cutaneous inflammatory mediator that is highly effective in suppressing several differentiation markers and in preserving proliferative potential of keratinocytes. Exposure to sodium vanadate in the medium greatly prolonged the phosphorylation of STAT1, but only at high concentration (>30 µM). Inhibitors of protein tyrosine phosphatases known to dephosphorylate STAT1 (SHP2, TCPTP, PTP1B) were ineffective in mimicking the action of vanadate. The irreversible protein tyrosine phosphatase inhibitor phenyl vinyl sulfonate alone induced STAT1 phosphorylation and appeared to induce its limited cleavage. It also inhibited cross-linked envelope formation, a characteristic step of keratinocyte terminal differentiation, likely due to its reaction with the active site cysteine of keratinocyte transglutaminase. Thus, the key protein tyrosine phosphatase responsible for STAT1 dephosphorylation remains to be identified, and an off-target effect of a potential inhibitor was revealed.

## Introduction

The epidermis of human skin carries out numerous activities to maintain barrier function. Playing major roles in regulating these activities, cytokines participate in normal keratinocyte growth and differentiation, wound healing and immune system modulation. In situations where homeostasis goes awry, they also contribute to pathological states such as psoriasis, hypersensitization and cancer (Noske, 2018). These diseases become manifest through genetic and environmental factors often involving complex feedback loops (Albanesi et al., 2018). In addition to responding to the cytokines they encounter in their microenvironment, keratinocytes participate in inflammatory responses through secretion of a repertoire of cytokines and related effectors.

A major pathway by which cytokine, growth factor and G-protein coupled receptors signal is through the Janus tyrosine kinases that, among other things, activate DNA binding proteins such as signal transducers and activators of transcription (STATs). Our previous work in cultured human epidermal keratinocytes showed that the cytokine IL4 induces dual oxygenases 1 and 2, while interferon-gamma induces only dual oxygenase 2 (DUOX2) (Hill III & Rice, 2018). In both cases, DUOX2 induction was markedly augmented by sodium vanadate concentrations as low as 5 µM. The action of vanadate, presumably as an inhibitor of protein tyrosine phosphatase activity (PTP) and at much higher concentration (Gordon, 1991), has been reported previously in fibroblasts to preserve the state of STAT1 phosphorylation and thus to maintain cytokine signaling (David et al., 1993; Haspel & Darnell, 1999). Elucidating the mechanism of vanadate activity could help clarify protein targets by which environmental factors can influence cytokine signaling in keratinocytes.

Oncostatin M (OSM), derived mainly from immune cells, has many functions in a variety of target tissues and can contribute to pathological processes in them (Hermanns, 2015). Transcriptional responses to OSM indicate this cytokine affects innate immunity, adhesion, motility, cell cycle and metabolism in cultured keratinocytes (Finelt et al., 2005). A member of the IL6 family, it is an important inflammatory mediator in the skin (Boniface et al., 2007) and is known to suppress differentiation in cooperation with other cytokines (Rabeony et al., 2014). It also promotes proliferation of certain tumor cell types and progression of cutaneous and squamous cell carcinomas (Simonneau et al., 2018). Like other cytokines, OSM signals through STAT1 and STAT3 as well as the mitogen-activated protein kinase pathway (Dey et al., 2013), where sustained phosphorylation on tyrosine residues helps maintain transcriptional activity (Andrés et al., 2013). Present work addresses possible effects of treatments modifying STAT phosphorylation that could alter OSM signaling.

We test whether OSM, similar to epidermal growth factor (Patterson & Rice, 2007), preserves keratinocyte proliferative potential while suppressing differentiation and whether vanadate maintains OSM signaling in keratinocytes as judged by STAT1 phosphorylation on tyrosine 701. Since vanadate was effective only at high concentration, the relative effectiveness of several other PTP inhibitors was assessed. Treatment of the cells by one of these, phenyl vinyl sulfonate (PVSN), induced STAT1 activation, even in the absence of OSM. In addition, it exhibited the unanticipated property of inhibiting keratinocyte cross-linked envelope formation, likely due to reactivity with enzyme active site cysteines, in this case of keratinocyte transglutaminase (TGM1).

## Materials & Methods

### Materials

Phenyl vinyl sulfonate and phenyl vinyl sulfone (PVS) were purchased from Enamine Ltd (Monmouth, NJ). JTT-551 (PTP1B and TCPTP inhibitor; IC50 = 0.2 and 2 µM, respectively), NSC-87877 (SHP-1 and SHP-2 inhibitor; IC50 = 0.36 and 0.32 µM, respectively), and CX08005 (PTP1B and TCPTP inhibitor; IC50 = 0.78 and 0.48 µM, respectively) were obtained from Millipore Sigma (Darmstadt, Germany). *β*-actin mouse monoclonal antibody (clone AC-74, catalog number A5316) was obtained from Sigma-Aldrich (St. Louis, MO). STAT1 (rabbit monoclonal, catalog number 9175), phospho-STAT1 (tyrosine 701, rabbit monoclonal, catalog number 9167), phospho-STAT3 (tyrosine 705, rabbit monoclonal, catalog number 9131), Caspase-3 (rabbit monoclonal, catalog number 14220), anti-biotin HRP linked (goat, catalog number 7075), anti-rabbit (polyclonal) and anti-mouse (polyclonal) antibodies and cytokines were purchased from Cell Signaling Technology (Danvers, MA). Sodium vanadate was dissolved in water, sterile filtered and stored at 4 °C until use (Hill III & Rice, 2018).

### Cell culture

Spontaneously immortalized human epidermal keratinocytes (SIK) (Rice et al., 1993) were grown in a mixture of Dulbecco’s modified Eagle’s and F12 media (2:1) containing fetal bovine serum (5%), hydrocortisone (0.4 µg/ml), adenine (0.18 mM), transferrin (5 µg/ml) and insulin (5 µg/ml) and co-cultured with a feeder layer of lethally irradiated 3T3 cells (Allen-Hoffmann & Rheinwald, 1984). The medium was supplemented with cholera toxin (10 ng/ml) (EMD Biosciences, La Jolla, CA) at inoculation and with epidermal growth factor (10 ng/ml) at the first and subsequent medium changes. The medium was changed at 3 to 4-day intervals. For inhibitor treatments, cultures (≈90% confluent) were treated with the indicated PTP inhibitor for given times, the medium containing the inhibitor was removed, and OSM was added in prewarmed fresh medium at 50 ng/ml for indicated times.

### Differentiation and colony forming efficiency measurements

Newly confluent cultures were treated with 10 ng/ml IL4, IL6, IL20, or 50 ng/ml OSM. These commonly used concentrations were confirmed to give roughly equivalent phosphorylation of STAT1 in the SIK cultures after one hr of treatment. For measurement of differentiation markers, cells were dissolved in Trizol reagent (Invitrogen) after 3–6 days of treatment, and cDNA was synthesized using high-capacity cDNA Reverse Transcription kit (Applied Biosystems). mRNA levels were measured using real-time PCR with TaqMan Fast PCR Master Mix, TaqMan Gene Expression assays (Applied Biosystems) and ABI 7500 Fast Sequence Detection System. Relative mRNA levels were calculated after normalization of each sample to MAPK1 mRNA. Colony forming efficiency was measured after 10 days of treatment. To this end, 3,000 cells were passaged in 6-cm dishes with a lethally irradiated 3T3 feeder layer, grown until colonies were large enough to see easily (7–14 d), fixed and stained with 0.1% rhodamine in water for counting.

### Western blotting

Cultures were harvested into a buffer containing 2% w/v SDS, 62.5 mM Tris (pH 6.8), 10% glycerol, with added 5 mM sodium pyrophosphate and 50 µM sodium vanadate to inhibit phosphatase activity. Samples were boiled for 5–7 min and sonicated. Protein was measured with the bicinchoninic acid (Pierce Rockford, IL) (Smith et al., 1985) before incubation with 20 mM dithiothreitol for 20 min at 37 °C. Equal amounts of protein (16–20 µg) were separated by 10% SDS polyacrylamide gel electrophoresis and transferred to Immobilon membranes (Millipore, Danvers, MA). The membranes were blocked with 5% dry milk in Tris-buffered saline and 0.05% Tween 20, followed by incubation with primary antibodies and horseradish peroxidase-conjugated secondary antibodies. Immunopositive bands were detected using ECL Plus Chemiluminescence detection reagent (Pierce, Rockford, IL), detected and quantitated using a Thermo myECL imager (Waltham, MA). Exposure time for each experiment ranged between 1 to 44 min. *β*-actin served as a loading control.

### Assay for transglutaminase activity

Cultures were harvested at confluence into a buffer containing 10 mM Tris (pH 8), 1 mM EDTA and immediately sonicated. Inhibitors and 3 mM CaCl_2_ were incubated for 10 min at 37 °C with aliquots of cell sonicate, followed by 2 mM 5-(biotinamido)pentylamine (Lee et al., 1992) for 30 min. Samples were suspended in SDS buffer containing 20 mM dithiothreitol, 20 µg of protein were subjected to SDS polyacrylamide gel electrophoresis and transferred to Immobilon membranes. The membranes were blocked with 5% dry milk in Tris-buffered saline and 0.05% Tween 20, followed by incubation with HRP linked anti-biotin. Immunopositive bands were detected using ECL Plus Chemiluminescence detection reagent and quantitated (Johnson et al., 2012) using a Thermo myECL imager with Thermo My Image Analysis software. Band values were normalized to those from the untreated samples.

### Statistical analysis

Statistical significance (*p* = 0.05) was calculated by one-way ANOVA (Stata/SE9.2 software for Windows) or two-way ANOVA (Wessa, 2020) with Tukey HSD testing.

## Results

### Striking keratinocyte responses to OSM and representative cytokines

**Figure 1:**
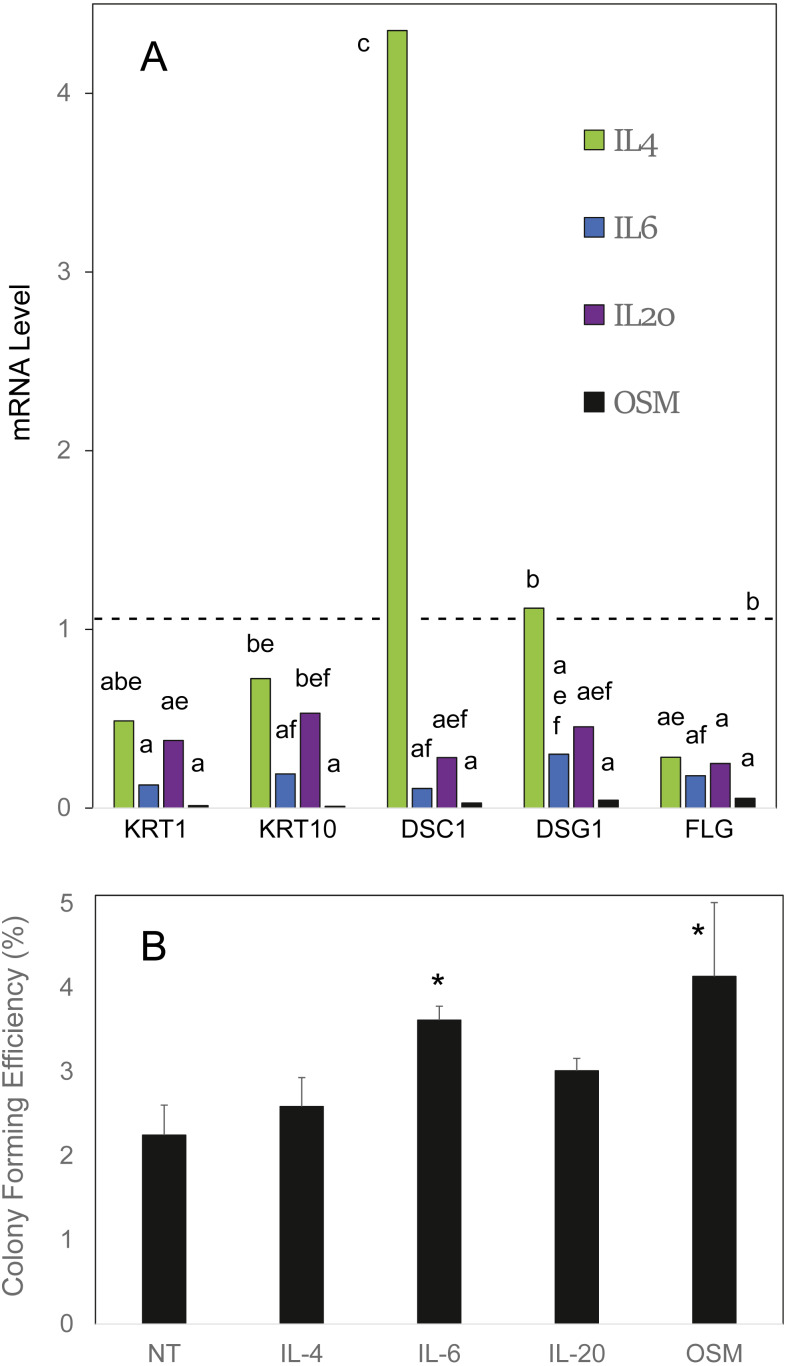
Cellular responses to cytokine treatment. (A) Cultures treated for 3 days with the indicated cytokines were analyzed by real time PCR for relative expression of keratin 1 (KRT1), keratin 10 (KRT10), desmocollin-1 (DSC1), desmoglein-1 (DSG1) and filaggrin (FLG). mRNA levels were normalized to mitogen-activated protein kinase-1 and reported relative to untreated cultures. Samples whose levels were not significantly different from each other (*p* = 0.05) share the same label (A–F). (B) Cultures treated for 10 days with the indicated cytokine were passaged at low density for measurement of colony forming efficiency. Statistical significance was calculated by two-way ANOVA for two independent experiments (A) or one-way ANOVA for four independent experiments (B).

OSM was compared to the cytokines IL4, IL6 and IL20 in effects on cultured human epidermal cells. As shown in Fig. 1A, IL6 and OSM each suppressed the differentiation markers keratin 1, keratin 10 and filaggrin. In contrast to IL4, which was markedly stimulatory toward desmocollin 1, IL6, IL20 and OSM suppressed expression of the junctional proteins desmocollin 1 and desmoglein 1. In each case, OSM produced a striking degree of suppression. When the cultures were treated for 10 days starting at confluence, at which time colony forming ability markedly declines (Patterson et al., 2005), OSM and IL6 partially preserved the cellular colony forming ability (Fig. 1B), evidently slowing the departure of stem-like cells from the germinative pool. The results with IL4 and IL20 also displayed a trend (not statistically significant) toward this effect. OSM was employed for the succeeding experiments.

### Vanadate maintenance of STAT1 phosphorylation only at high concentration

**Figure 2:**
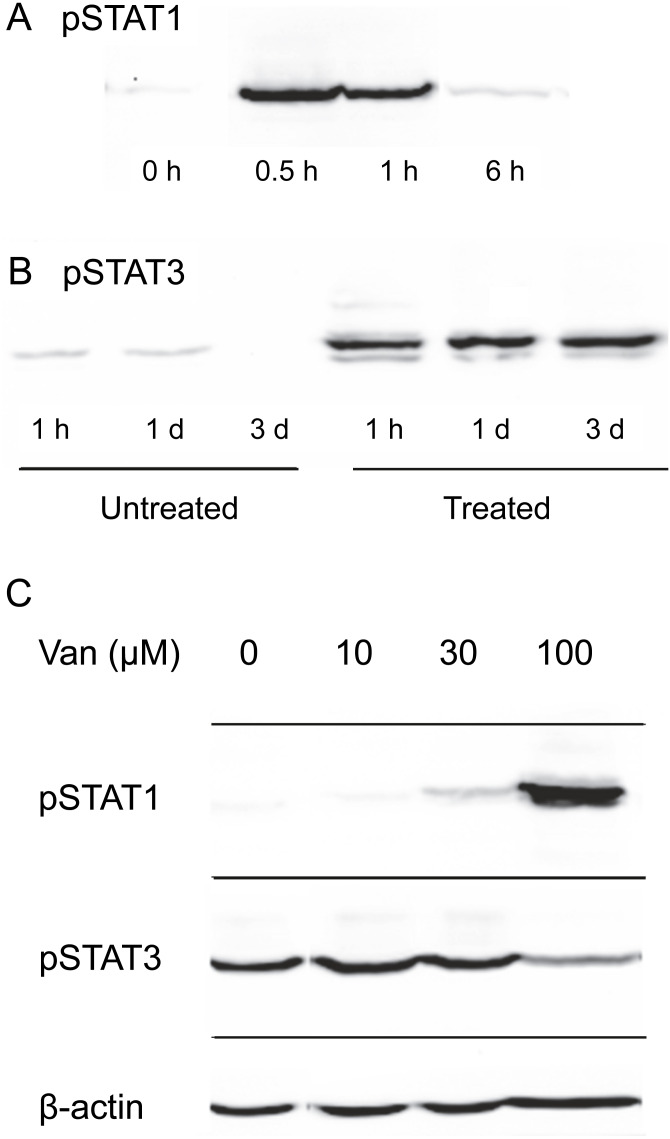
Dependence of OSM-induced STAT1 and STAT3 phosphorylation on vanadate concentration. Time-dependence of OSM-induced (A) phospho-STAT1 and (B) phospho-STAT3 accumulation. Cultures were treated with OSM for the indicated times prior to harvesting and western blotting. (C) Cultures were treated with OSM and vanadate concentrations as indicated, harvested after 24 h and submitted for western blotting with anti-phospho-STAT1, anti-phospho-STAT3 or anti-*β*-actin (loading control). Total STAT levels were not analyzed in this experiment. Two independent experiments were performed.

Induction of STAT1 phosphorylation on tyrosine 701 by OSM was tested in SIK cultures. As shown in Fig. 2A, the stimulation was transitory, reaching a maximal level within the first hour of OSM treatment and returning close to background level by 6 h. This pronounced decline in phosphorylation was in contrast to the stability of the phosphorylation induced in STAT3, which remained near maximal level for three days (Fig. 2B).

To test the possibility that vanadate could augment cytokine signaling in keratinocytes by preserving STAT1 phosphorylation, SIK cultures were treated for one day in the presence of various concentrations of sodium vanadate and then for one day with OSM. As shown in Fig. 2C, little phosphorylation of STAT1 was detected from treatment for 24 hr with OSM alone. A detectable level of phosphorylation was seen in combination with 30 µM vanadate and a substantially higher level with 100 µM vanadate, while 10 µM vanadate was ineffective. Vanadate alone was also ineffective, and when cultures were treated with 100 µM vanadate for only two hr, followed by a medium change and treatment with OSM for 6 hr, STAT1 phosphorylation was not augmented (Fig. S1).

### Selective PTP inhibitors did not prevent STAT1 dephosphorylation

**Figure 3:**
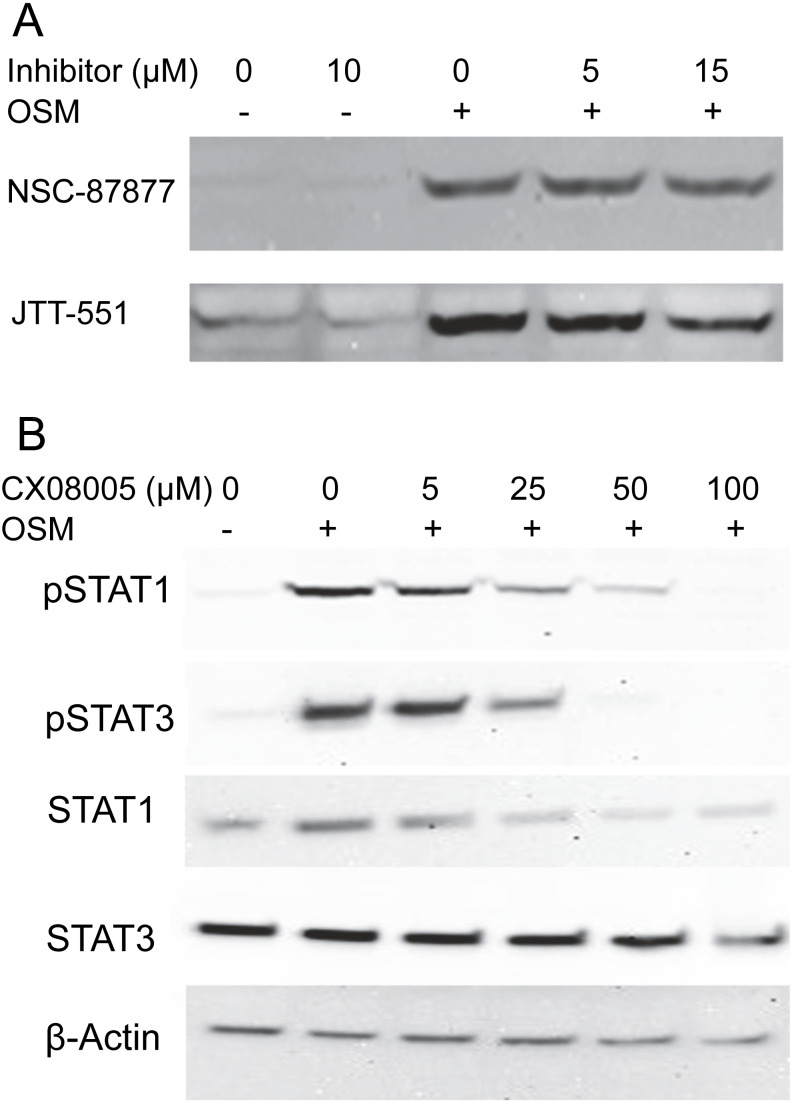
Protein tyrosine phosphatase inhibitors not augmenting phosopho-STAT1 levels. (A) Cultures were treated with the indicated inhibitor for 1 h and then with OSM for 1 day before harvesting and western blotting with anti-phosphoSTAT1 antibody. Medium concentrations of each inhibitor were well above the IC50 for JTT-551 (0.2 µM for PTP1B) and NSC-87877 (0.3–0.4 µM for SHP-1 and SHP-2). (B) Cultures were treated with CX08005 at the indicated concentrations for two hr and harvested after 6 h. Medium concentration of the inhibitor was well above the IC50 (0.48 and 0.78 µM for TCPTP and PTP1B, respectively). Two independent experiments were performed.

To gain information about the hypothetical PTP activity responsible for reversing the cytokine-induced phosphorylation of STAT1, cultures were treated with OSM alone or in the presence of the inhibitors NSC-87877, a selective inhibitor of SHP2 (Chen et al., 2006), or JTT-551, a known inhibitor of PTP1B (Fukuda et al., 2010). The inhibitors neither affected the level of phospho-STAT1 when administered alone nor gave higher levels of phospho-STAT1 when administered with OSM. Figure 3A shows the lack of effect at concentrations an order of magnitude higher than the IC50s toward PTP1B or SHP-1 and SHP-2. Although TCPTP has been reported to dephosphorylate STAT1 in HeLa cells (ten Hoeve et al., 2002), increasing inhibitor concentrations to 100 µM in SIK did not affect the result (Fig. S2). In addition, when administering CX08005, a more potent competitive inhibitor for PTP1B and TCPTP (Zhang et al., 2016) at various concentrations with OSM, both phospho-STAT1 and phospho-STAT3 were suppressed (and total STAT1 appeared reduced) at the highest concentration (Fig. 3B). The results provide no support for SHP2, TCPTP or PTP1B affecting phospho-STAT1 signaling in SIK cultures by OSM.

### Off-target effects of phenyl vinyl sulfonate and phenyl vinyl sulfone

**Figure 4:**
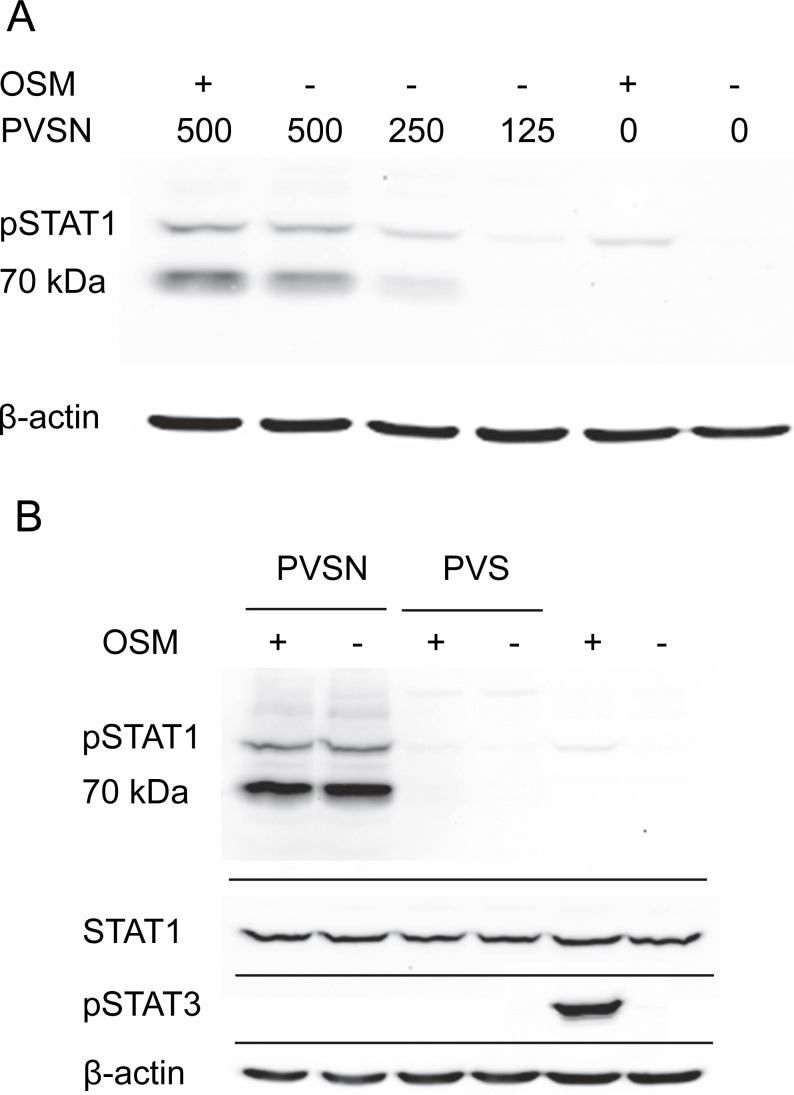
PVSN-induced STAT1 phosphorylation with and without OSM treatment. (A) Cultures were treated with the inhibitor at the indicated concentrations (µM) for two hr and harvested after 6 h. Total STAT1 levels were not analyzed. (B) Cultures were treated with 500 µM inhibitor and OSM as indicated and examined for phospho-STAT1 (pSTAT1) and phospho-STAT3 (pSTAT3). Three independent experiments were performed.

Two members of a more general class of PTP inhibitor were examined for their effect on STAT1 phosphorylation. Aryl vinyl sulfonates and sulfones are directed to the active site sulfhydryls in these phosphatases (Liu et al., 2008). Phenyl vinyl sulfone treatment had little effect, but phenyl vinyl sulfonate (PVSN) induced a striking increase in phospho-STAT1 in the absence of OSM treatment as shown in Fig. 4A. The effect was maximal at 500 µM in the presence or absence of OSM treatment, and considerably lower at 250 µM. By contrast, PVS had little effect on phospho-STAT1 level (Fig. 4B).

In addition to an increase in phospho-STAT1 (90–100 kDa), Fig. 4B showed an even more strongly immunoreactive band at 70–80 kDa detected by the antibody staining. Moreover, in the presence of either PVSN or PVS, the band of phospho-STAT3 seen with OSM treatment alone was eliminated. The appearance of phospho-STAT1 with altered mobility could plausibly result from limited proteolysis by caspase-3 (King & Goodbourn, 1998), an activity that might degrade phospho-STAT3. Cell extracts were therefore examined for activated caspase-3, a potential source of proteolytic activity and an indicator of apoptosis, but only procaspase-3 was detected (Fig. S3).

### Prevention of keratinocyte transglutaminase cross-linking

**Figure 5:**
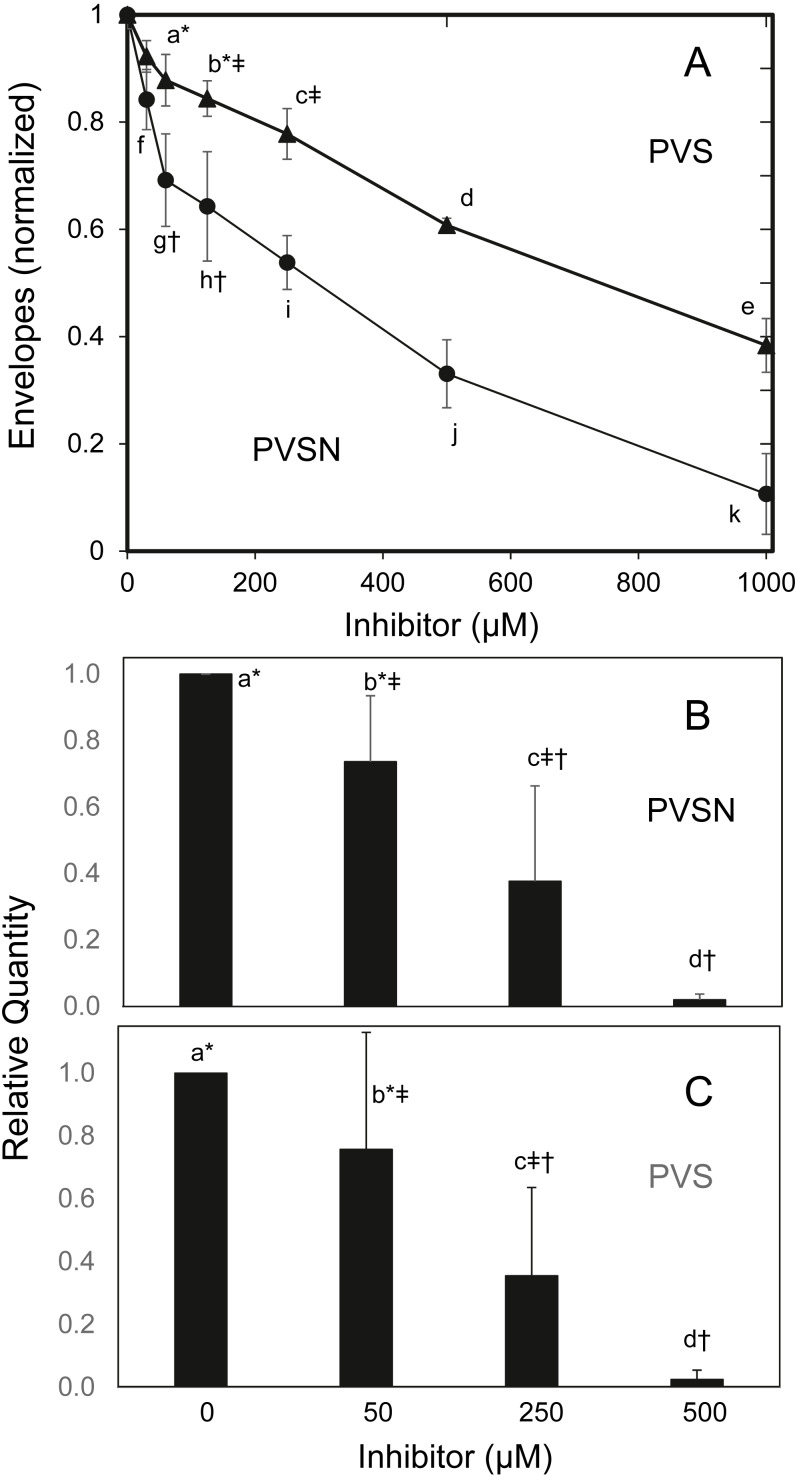
Inhibition of cross-linked envelope formation and protein biotinylation by protein tyrosine phosphatase inhibitors. (A) After two hr incubation with the inhibitors, the medium was replaced by serum free medium containing X537A (70 µM), activating TGM1. Cultures were incubated overnight, and then suspended in 1% SDS—10 mM dithiothreitol—0.05 M Tris buffer (pH 8) for several hours. Envelopes were recovered by centrifugation, rinsed four times in 0.1% SDS and quantitated by protein assay using bicinchoninic acid. Values with different letters were significantly different from each other except the pairs indicated by symbols (*, †, ‡) as judged by two-way ANOVA in three independent trials (each in triplicate).Culture extracts treated with biotinamido-pentylamine and (B) PVSN or (C) PVS were immunoblotted with anti-biotin. Total biotinylated protein was quantitated. Values with different letters were significantly different from each other except the pairs indicated by symbols (*, †, ‡) by one-way ANOVA in three independent experiments.

To find whether the cultures survived treatment with PVSN, cells were tested for formation of cross-linked envelopes, a distinctive feature of keratinocyte terminal differentiation that occurs when the plasma membrane becomes permeable at cell death (Green, 1979). At the most effective inhibitor concentration for induction of STAT1 phosphorylation (500 µM), few spontaneous envelopes were observed as a consequence of treatment. To check whether the cells were capable of envelope formation, cultures were treated with the ionophore X537A, known to induce envelope formation in a large majority of keratinocytes in such cultures (Rice & Green, 1979). As shown in Fig. 5A, ionophore-induced envelope formation was inhibited in a concentration-dependent manner. PVS was also effective but less potent, as shown, whereas vanadate (120 µM), NSC-87877 (20 µM) and JTT-551 (20 µM) neither prevented (Fig. S4) nor induced envelope formation under the same conditions.

TGM1, which forms envelopes by cross-linking proteins at the plasma membrane through isopeptide bonds (Rice & Green, 1979), can be assayed by measurement of aliphatic amine incorporation into protein substrates. To confirm TGM1 was inactivated by PVSN and PVS treatment, incorporation of a *biotinylated amine* transglutaminase substrate was measured in cell extracts. As shown in Fig. 5B, total biotinylated protein was lowered in both PVSN and PVS treated cell extracts at increasing concentration and essentially prevented at 500 µM. While exhibiting similar potencies in cell extracts, the inhibitors could differ in potencies of suppressing envelope formation due to different rates of entry into cells.

## Discussion

Our previous results showed that vanadate noticeably stimulated DUOX2 induction in cultured keratinocytes by IL4 and IFN*γ* at concentrations as low as 5 µM. We explored whether vanadate action was mediated by stabilization of STAT1 phosphorylation through inhibition of a protein tyrosine phosphatase as shown by others in fibroblasts (David et al., 1993; Haspel & Darnell, 1999). However, present work shows that vanadate was at least 100 fold less potent in preserving OSM-induced STAT1 phosphorylation of tyrosine 701 than it had been in stimulating DUOX2 induction. Efforts to demonstrate that inhibition of PTP activity preserved the phosphorylation using specific inhibitors of certain PTPs expressed in keratinocytes were not successful, although inhibition of only a small number of those expressed in SIK (Table S1) could be tested. We note that use of such inhibitors alone is insufficient to identify a responsible phosphatase, since their specificity may depend on target species and cellular context. For example, NSC-87877 is reportedly ineffective toward SHP2 in certain cell lines (Tsutsumi, Ran & Neel, 2018). If any inhibitors had shown activity, further efforts to validate their actions would have been required.

This work illustrates well the phenomenon of off-target effects of inhibitors. Use of PVSN, an irreversible inhibitor of PTPs that targets their active site cysteine residues, resulted in increased levels of STAT1 phosphorylation. However, since the increase was not dependent on added cytokine, the mechanism appeared indirect. Moreover, although immunoblotting showed a STAT1-immunopositive band at the expected mobility of 90–100 kDa, a more intense immunopositive band at 70–80 kDa was also evident, suggesting that STAT1 was subject to limited proteolytic cleavage. Induction of apoptosis or terminal differentiation by the treatment would be consistent with STAT1 cleavage by caspase-3 (King & Goodbourn, 1998), but activation of this caspase was not detected. An alternate interpretation, supported by no observed change in total STAT1 immunoreactivity, is that PVSN induced a 70 kDa protein that was immunoreactive toward the anti-phosphoSTAT1 antibody employed.

Furthermore, this class of inhibitor appeared not to be specific for PTPs. As a consequence, testing whether protein tyrosine phosphatase inhibition with this reagent produced terminal differentiation as a result of treatment was inconclusive. Formation of cross-linked envelopes, a diagnostic feature of terminal differentiation (Green, 1979), was suppressed, but PVS and especially PVSN inhibited this process, likely by targeting the transglutaminase (TGM1) responsible for cross-linking at its active site cysteine. This inhibitor class differs from those previously investigated as transglutaminase inhibitors (Keillor, Apperley & Akbar, 2015; Song et al., 2017). Since inactivating mutations of TGM1 are the predominant cause of the skin disease autosomal recessive congenital ichthyosis (Pigg et al., 2016), this class of protein tyrosine phosphatase inhibitor would be inadvisable for therapeutic use in epidermis.

## Conclusion

The present study was based on the expectation that vanadate’s action in DUOX2 induction in human keratinocytes involved stabilization of STAT phosphorylation. If so, then it was expected to be much more potent in keratinocytes than previously reported in fibroblasts. However, testing this expectation gave a negative result. Subsequent efforts to stabilize STAT1 phosphorylation using protein tyrosine phosphatase inhibitors, and thereby to identify a responsible phosphatase, were undertaken and also proved negative. Consequently, that vanadate alters JAK/STAT signaling to promote induction of DUOX2 by must now be re-examined. IL4 and interferon-*γ* display differences in their mechanisms of DUOX induction in keratinocytes. For example, effectiveness of the latter, which does not act through JAK/STAT pathways (Hill III, Xu & Harper, 2010), is sensitive to quenching of reactive oxygen species (Hill III & Rice, 2018). Thus, if the sensitive target of vanadate in DUOX2 induction is a protein tyrosine phosphatase, it may not alter STAT phosphorylation. The possibility that a phosphatase targeted by vanadate could be identified by monitoring DUOX2 induction by IL4 merits further exploration. In any case, the activity responsible for STAT1 dephosphorylation in keratinocytes remains to be identified.

##  Supplemental Information

10.7717/peerj.9504/supp-1 Table S1 Relative mRNA levels of protein tyrosine phosphatases in SIK Normalized values were obtained from next generation sequencing (Phillips et al, 2016).

10.7717/peerj.9504/supp-2 Figure S1 Lack of vanadate action (0–100 µM) with 2 h treatment and removal with subsequent OSM treatment for 6 h As seen, vanadate alone was ineffective.

10.7717/peerj.9504/supp-3 Figure S2 Lack of effect of NSC-87877 (NSC) and JTT-551 (JTT) at 100 µM on level of pSTAT1

10.7717/peerj.9504/supp-4 Figure S3 Lack of caspase-3 activation detected by immunoblotting Cultures treated with PVS, PVSN and OSM as indicated were harvested and immunoblotted with anticaspase-3 and anti-*β*-actin as a loading control. For each lane in the lower panel, densities of the 40 and 34 kDa bands were quantitated, added and normalized to the density of the *β*-actin band. The values are shown in panel A.

10.7717/peerj.9504/supp-5 Figure S4 Lack of effect of phosphatase inhibitors on ionophore-induced envelope formation Cultures were treated for 2 hr. Envelopes were induced by treatment with X537A (70 µM) and the envelope protein was quantitated with bicinchoninic acid (A565).

10.7717/peerj.9504/supp-6 Figure S5 Images of original western blots for Figures 2–4 and S3 In each case, the image on the left shows the PageRuler prestained molecular weight markers (91, 86, 84, 45, 35 kDa). Indicated by the asterisk is the molecular weight corresponding to the protein of interest, and the image on the right shows the corresponding antibody staining.

10.7717/peerj.9504/supp-7 Figure S6 Replicate western blots of STAT1 Original images for Figures 2–4.

10.7717/peerj.9504/supp-8 Data S1 Figure S1 Data

10.7717/peerj.9504/supp-9 Data S2 Raw data for Figure 1

10.7717/peerj.9504/supp-10 Data S3 Raw data for Figure 5

## Abbreviations

DUOX2: dual oxygenase 2
OSM: oncostatin M
pSTAT: phospho-STAT
PTP: protein tyrosine phosphatase
PVS: phenyl vinyl sulfone
PVSN: phenyl vinyl sulfonate
SDS: sodium dodecyl sulfate
SIK: spontaneously immortalized keratinocytes
STAT1: signal transducer and activator of transcription 1
TGM1: keratinocyte transglutaminase

## Additional Information and Declarations

### Data Availability

The following information was supplied regarding data availability: The raw data and uncropped western blots are available in the Supplemental Files.
