# Supplementary material for: Off-target effects of protein tyrosine phosphatase inhibitors on oncostatin M-treated human epidermal keratinocytes: the phosphatase targeting STAT1 remains unknown
Source: PeerJ. 2020 Aug 14;8:e9504. doi: 10.7717/peerj.9504 (PMC7430265; doi:10.7717/peerj.9504)
Supplement: Table S1 — Normalized values were obtained from next generation sequencing (Phillips et al, 2016). [file peerj-08-9504-s001.pdf]

Table S1. Relative mRNA levels of protein tyrosine phosphatases in SIK.
Normalized values were obtained from next generation sequencing (Phillips et al, 2016).

PTP[1]	RPKM[2]	Features[3]
PTP4A1	11.9	prenylated
PTP4A2	24.8	prenylated
PTPDC1	1.4	centrosomal
PTPMT1	8.2	mitochondrial
PTPN1	21.4	*PTP1B[4]
PTPN11	25.7	*SHP2[4]
PTPN12	16.6	*PTPG1[4]
PTPN13	24.4	*
PTPN14	15.3	*
PTPN18	4.4	*
PTPN2	7.1	*TCPTP[4]
PTPN21	4.2	*
PTPN23	11.6	*
PTPN3	6.8	*
PTPN4	1.8	*
PTPN6	8.1	*SHP1[4]
PTPN9	3.0	*
PTPRA	15.0	
PTPRE	1.8	
PTPRF	82.7	
PTPRG	1.1	
PTPRK	6.3	
PTPRM	4.1	
PTPRS	3.0	
PTPRU	12.4	
PTPRZ1	3.4	

[1]Protein tyrosine phosphatase gene name
[2]Reads per kb per million mapped reads; limited to RPKM>1
[3]Lipid modification, localization or (*) non-receptor designation
[4]Alternate name
