# Supplementary material for: Off-target effects of protein tyrosine phosphatase inhibitors on oncostatin M-treated human epidermal keratinocytes: the phosphatase targeting STAT1 remains unknown
Source: PeerJ. 2020 Aug 14;8:e9504. doi: 10.7717/peerj.9504 (PMC7430265; doi:10.7717/peerj.9504)
Supplement: Figure S1 — As seen, vanadate alone was ineffective. [file peerj-08-9504-s002.pdf]

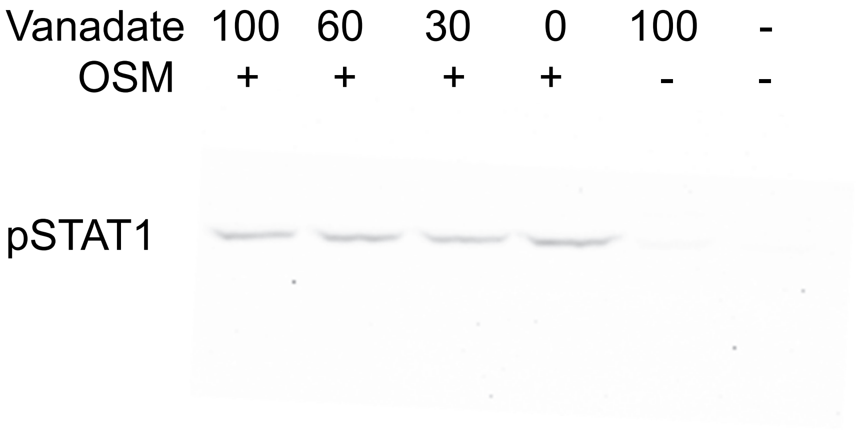

Fig S1. Lack of vanadate action (0-100 µM) with 2 hr treatment and removal with subsequent OSM treatment for 6 hr. As seen, vanadate alone was ineffective.
