# Supplementary material for: Off-target effects of protein tyrosine phosphatase inhibitors on oncostatin M-treated human epidermal keratinocytes: the phosphatase targeting STAT1 remains unknown
Source: PeerJ. 2020 Aug 14;8:e9504. doi: 10.7717/peerj.9504 (PMC7430265; doi:10.7717/peerj.9504)
Supplement: Figure S3 — Cultures treated with PVS, PVSN and OSM as indicated were harvested and immunoblotted with anticaspase-3 and anti-β-actin as a loading control. For each lane in the lower panel, densities of the 40 and 34 kDa bands were quantitated, added and normalized to the density of the β-actin band. The values are shown in panel A. [file peerj-08-9504-s004.pdf]

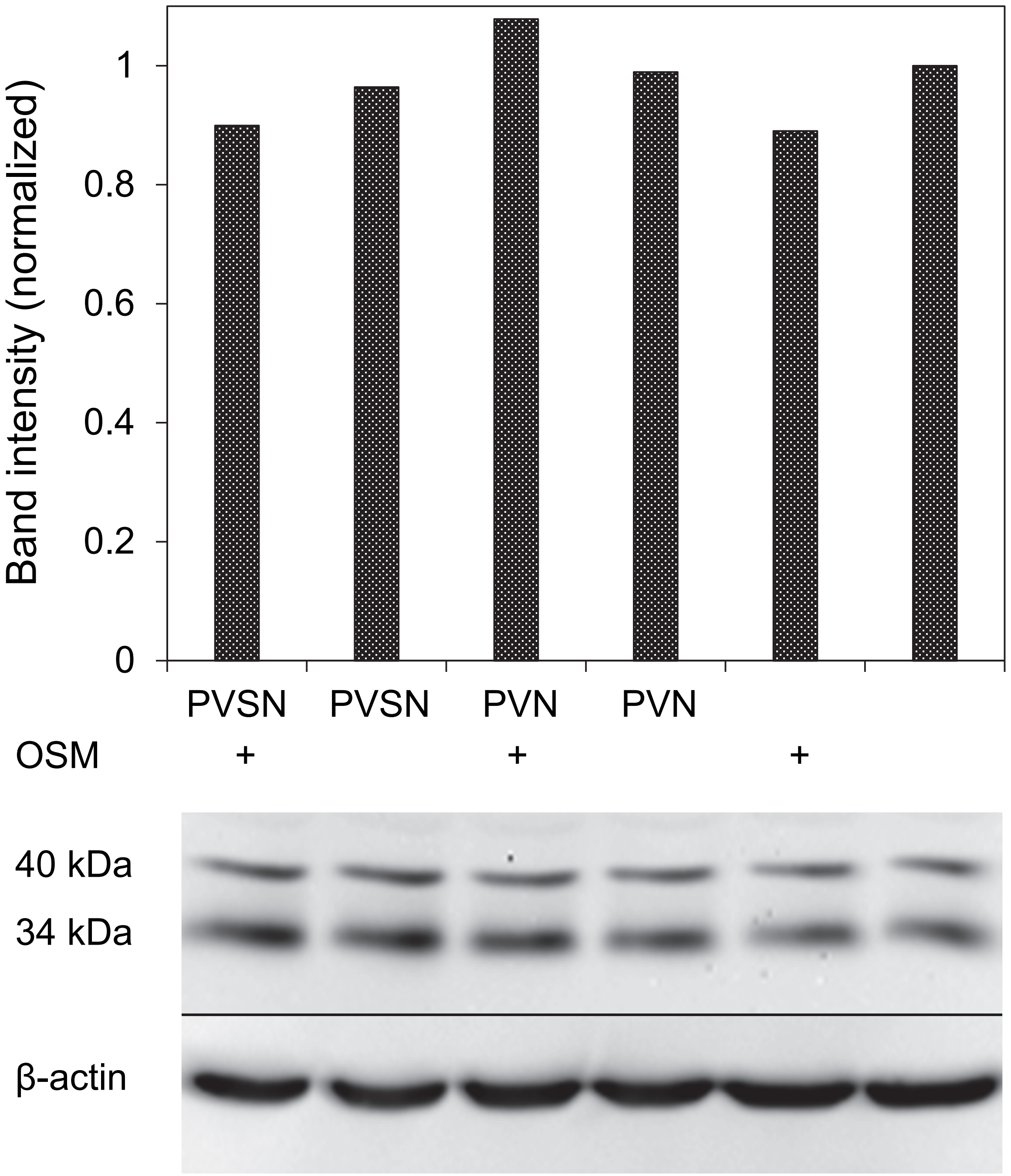

Figure S3. Lack of caspase-3 activation detected by immunoblotting. Cultures treated with PVS, PVSN and OSM as indicated were harvested and immunoblotted with anti-caspase-3 and anti-β-actin as a loading control. For each lane in the lower panel, densities of the 40 and 34 kDa bands were quantitated, added and normalized to the density of the β-actin band. The values are shown in panel A.
