# Supplementary material for: Off-target effects of protein tyrosine phosphatase inhibitors on oncostatin M-treated human epidermal keratinocytes: the phosphatase targeting STAT1 remains unknown
Source: PeerJ. 2020 Aug 14;8:e9504. doi: 10.7717/peerj.9504 (PMC7430265; doi:10.7717/peerj.9504)
Supplement: Figure S4 — Cultures were treated for 2 hr. Envelopes were induced by treatment with X537A (70 µM) and the envelope protein was quantitated with bicinchoninic acid (A565). [file peerj-08-9504-s005.pdf]

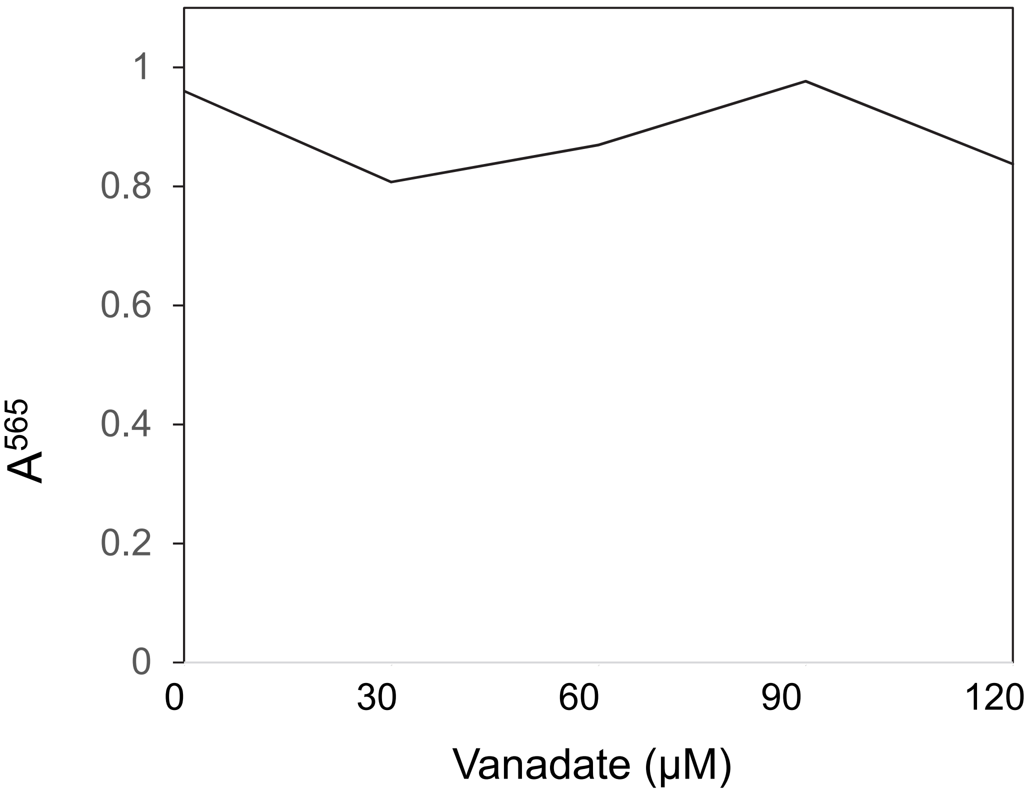

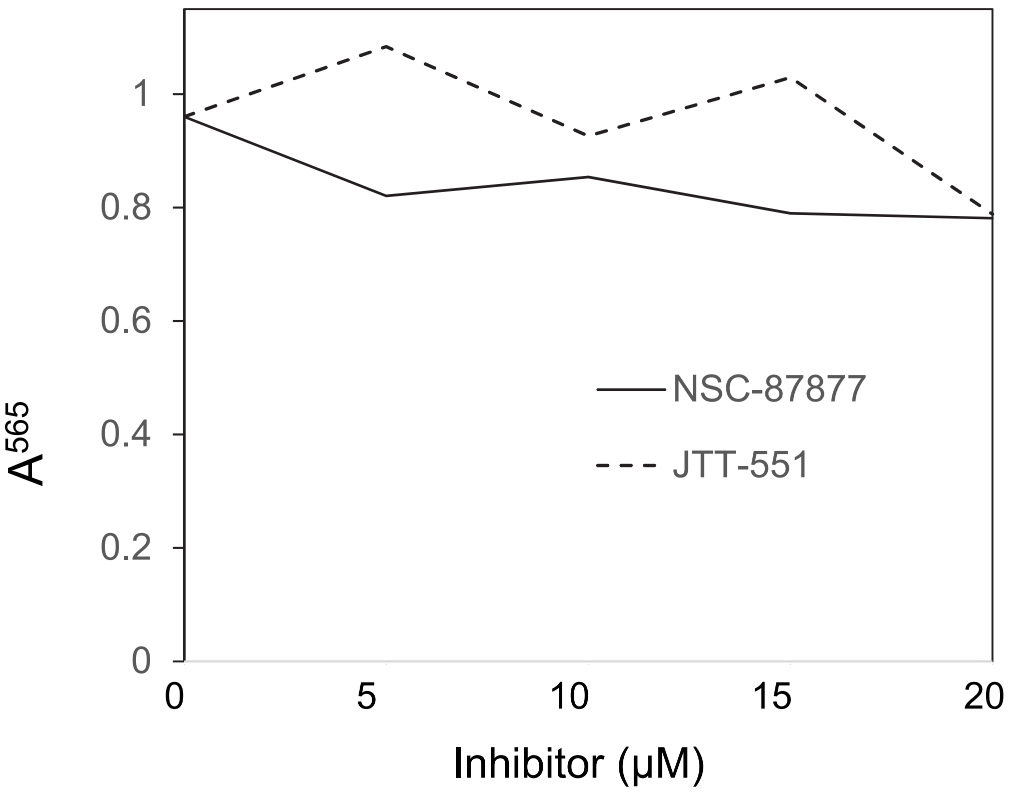

Figure S4. Lack of effect of phosphatase inhibitors on ionophore-induced envelope formation. Cultures were treated for 2 hr. Envelopes were induced by treatment with X537A (70 µM) and the envelope protein was quantitated with bicinchoninic acid (A^{565}).
