# Supplementary material for: Off-target effects of protein tyrosine phosphatase inhibitors on oncostatin M-treated human epidermal keratinocytes: the phosphatase targeting STAT1 remains unknown
Source: PeerJ. 2020 Aug 14;8:e9504. doi: 10.7717/peerj.9504 (PMC7430265; doi:10.7717/peerj.9504)

# Supplementary Figure S5

**Images of original western blots for Figures 2-4 and S3.** In each case, the image on the left shows the PageRuler prestained molecular weight markers (91, 86, 84, 45, 35 kDa). Indicated by the asterisk is the molecular weight corresponding to the protein of interest, and the image on the right shows the corresponding antibody staining.

**Fig. 2A Phospho-STAT1**

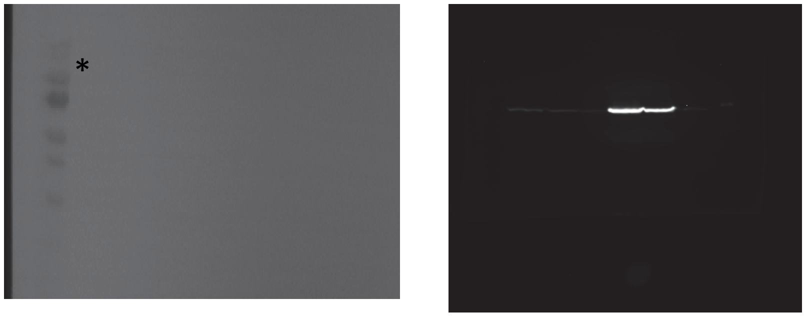

**Fig. 2B Phospho-STAT3**

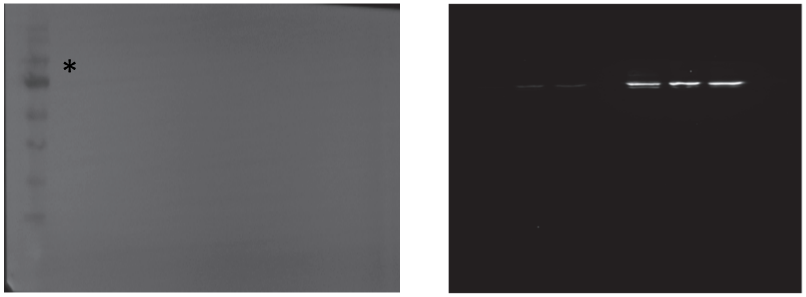

Fig. 2C
Phospho-
STAT1

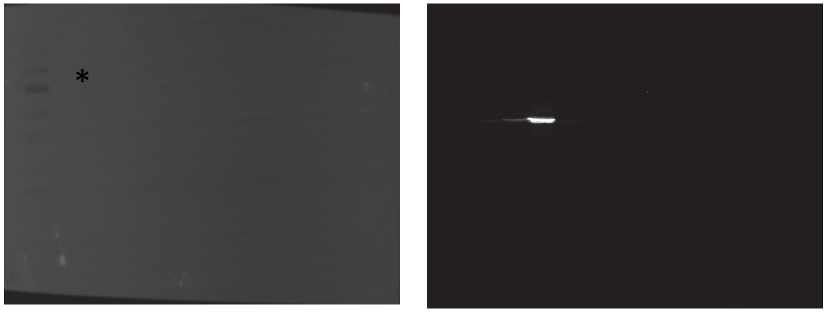

Fig. 2C
Phospho-
STAT3

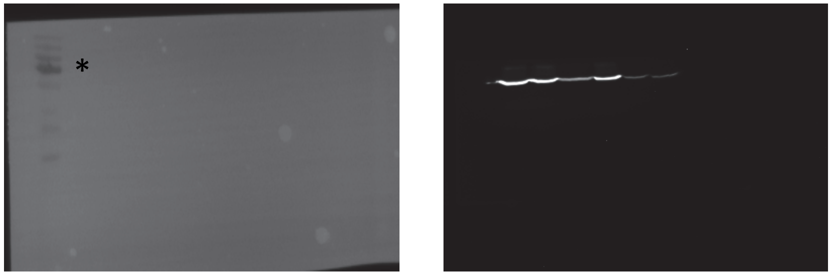

Fig. 2C
B-actin

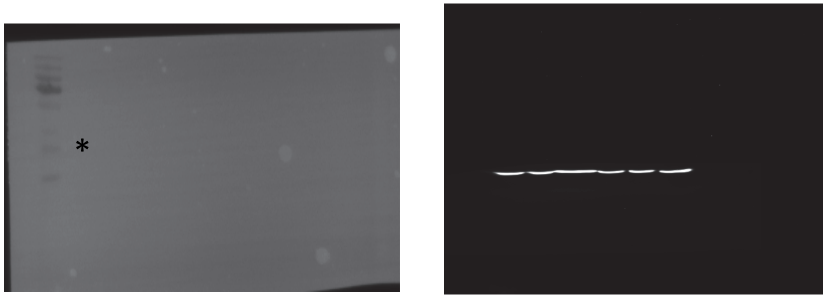

**Fig. 3A (JTT-551) phospho-STAT1**

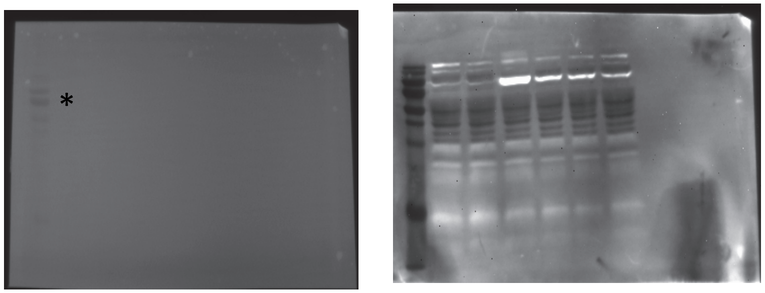


**Fig. 3A (NSC-87877) phospho-STAT1**

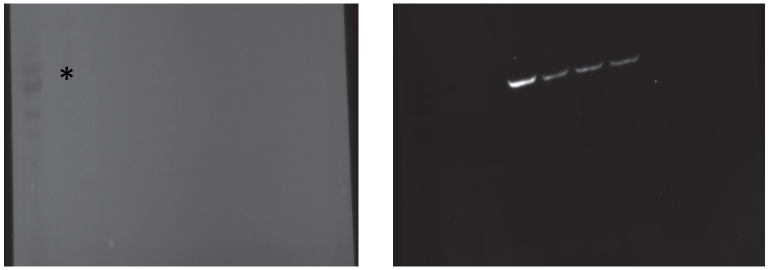

**Fig. 3B Phospho-STAT1**

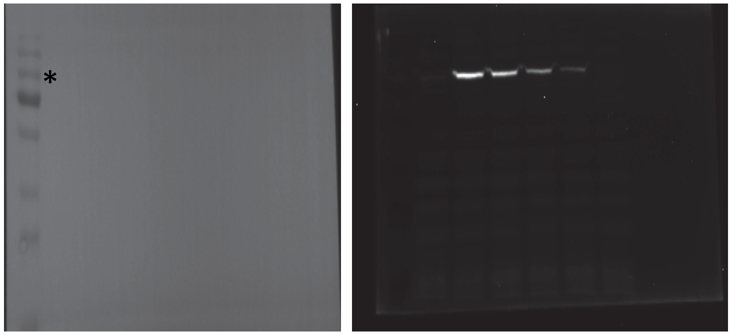

**Fig. 3B STAT1**

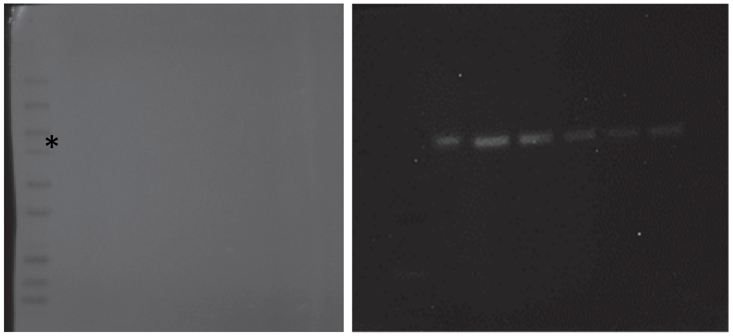

**Fig. 3B β-Actin (Lower Band)**

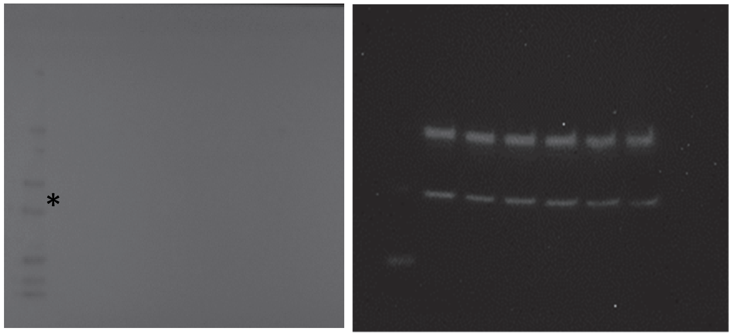

**Fig. 3B Phospho-STAT3**

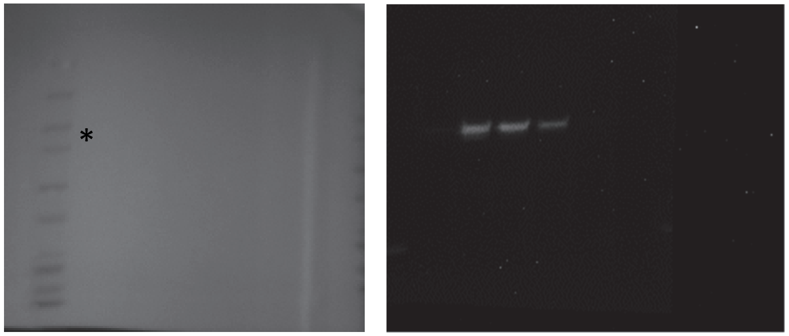

**Fig. 3B STAT3**

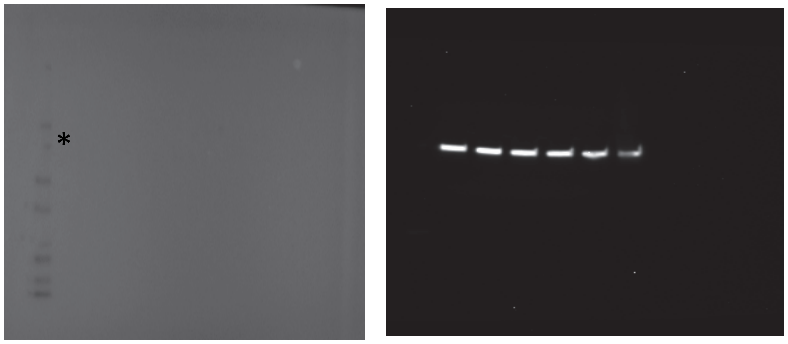

**Fig. 4A**
**Phospho-STAT1**

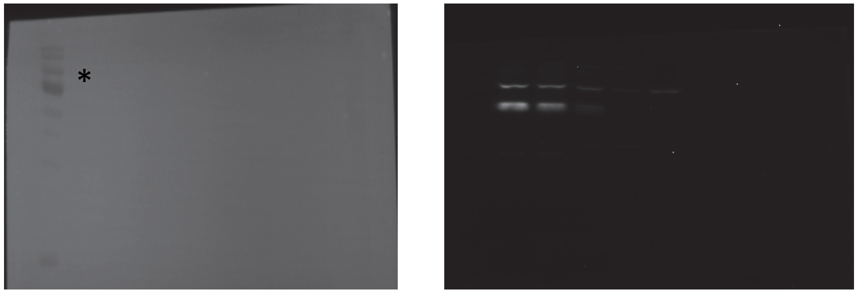


**Fig. 4A**
**β-Actin**

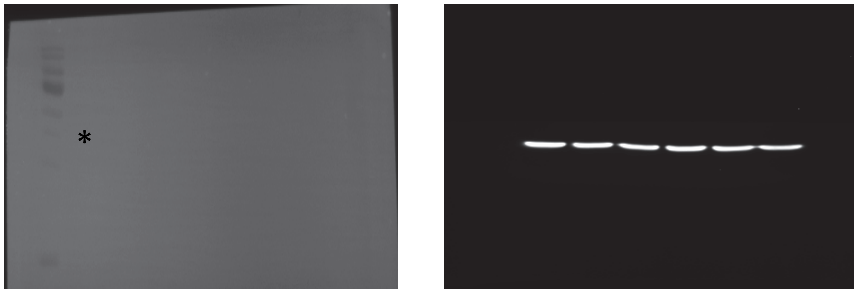

**Fig. 4B**
**Phospho-STAT1**

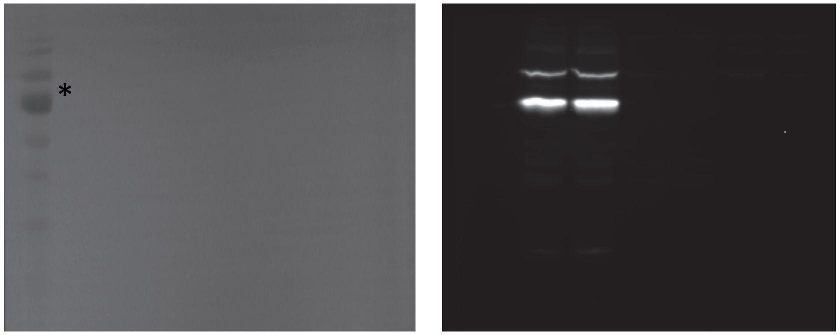

**Fig. 4B**
**Phospho-STAT3**

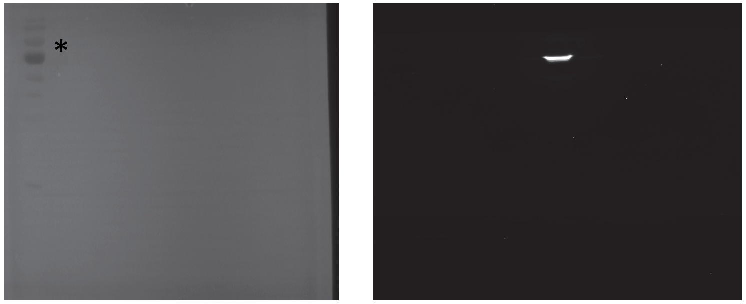

**Fig. 4B**
**STAT1**

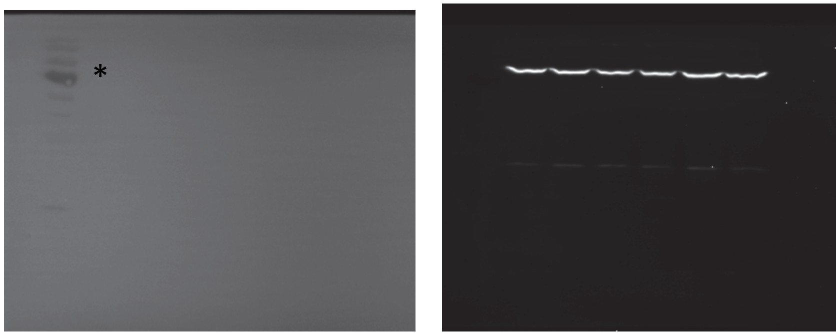

**Fig. 4B**
**β-Actin**

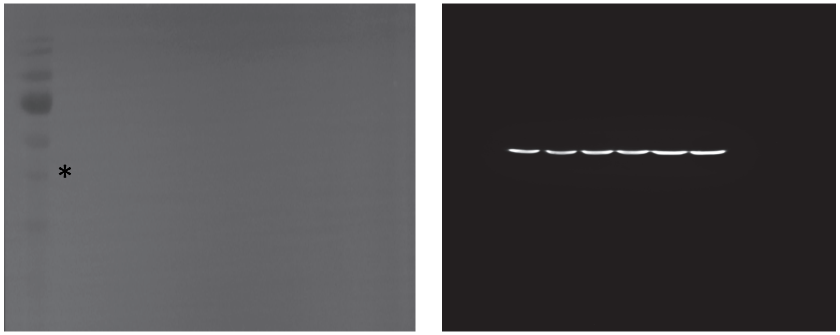

**Fig. S3**
**Caspase-3**

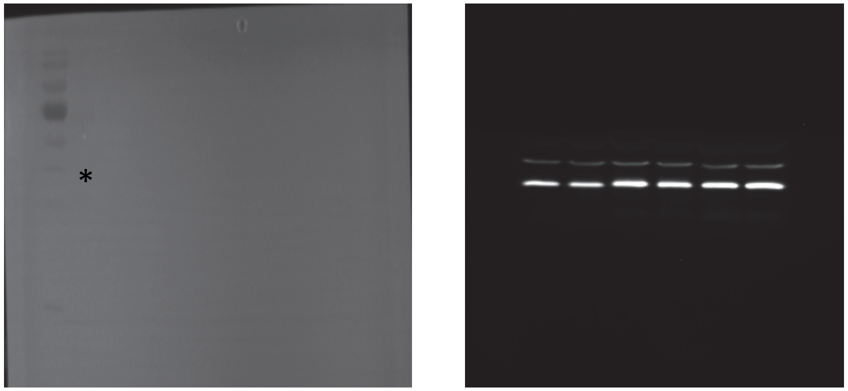

**Fig. S3**
**β-Actin**

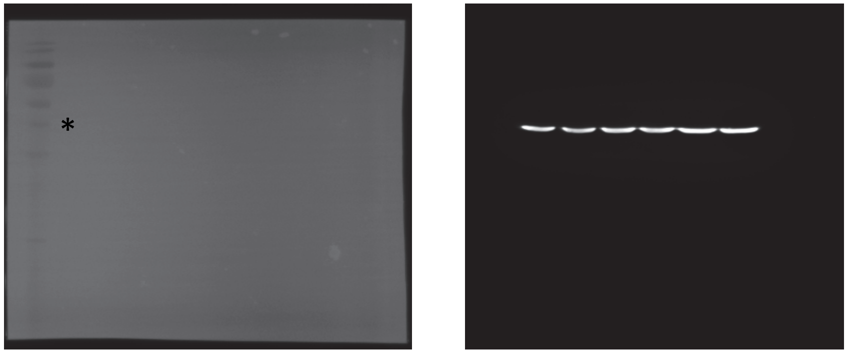

Supplement: Figure S5 — In each case, the image on the left shows the PageRuler prestained molecular weight markers (91, 86, 84, 45, 35 kDa). Indicated by the asterisk is the molecular weight corresponding to the protein of interest, and the image on the right shows the corresponding antibody staining. [file peerj-08-9504-s006.pdf]
