# Supplementary material for: Off-target effects of protein tyrosine phosphatase inhibitors on oncostatin M-treated human epidermal keratinocytes: the phosphatase targeting STAT1 remains unknown
Source: PeerJ. 2020 Aug 14;8:e9504. doi: 10.7717/peerj.9504 (PMC7430265; doi:10.7717/peerj.9504)

Supplementary Figure S3 - Replicate Western Blots of STAT1 – Original Images Figs 2-4

Figure 2A

OSM	+	+	+	-
OSM (ng/ml)	50	50	50	50
Time (hr)	6	0.5	1	0

pSTAT1 ➔

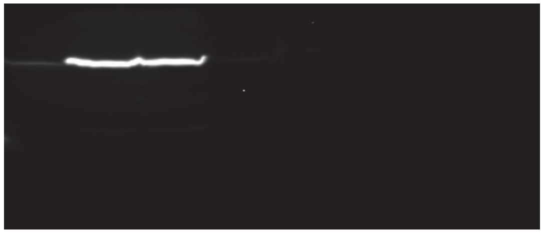

Replicate 2

Figure 2C

Replicate 2

OSM (50ng/ml)	+	+	+	+	-	-
Vanadate (µM)	10	30	100	0	30	0

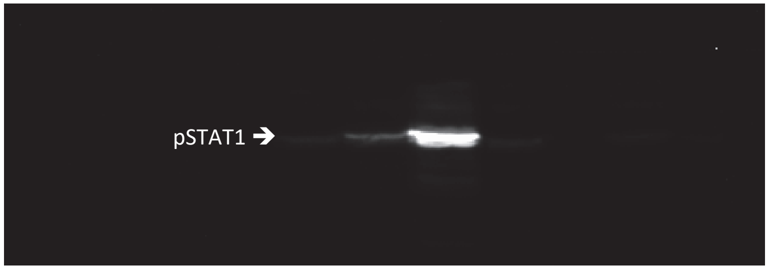

Replicate 3

OSM (50ng/ml)	+	+	+	+	-	-
Vanadate (µM)	10	30	100	0	30	0

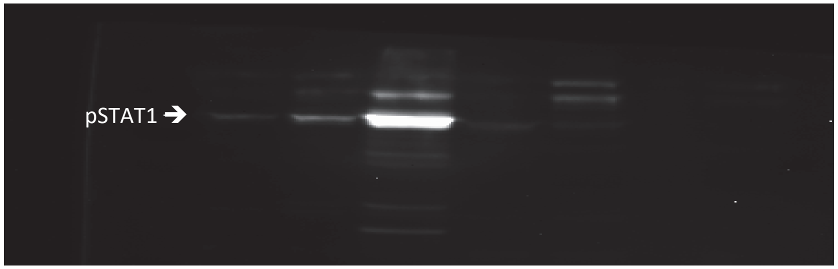

Figure 3A

Replicate 2

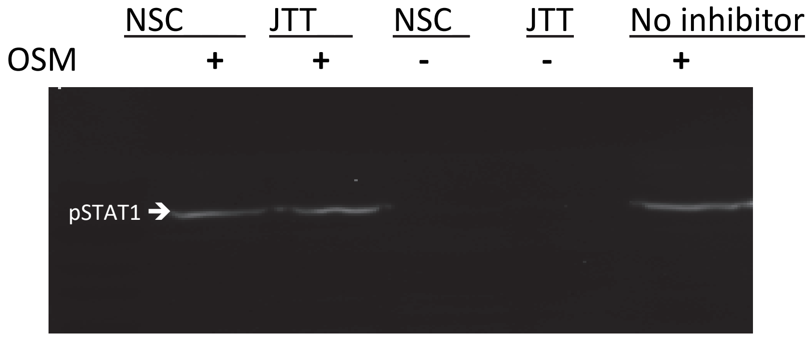

NSC-87877 and JTT-551 treated at 100 μM

Figure 3B

Replicate 2 with inverted image

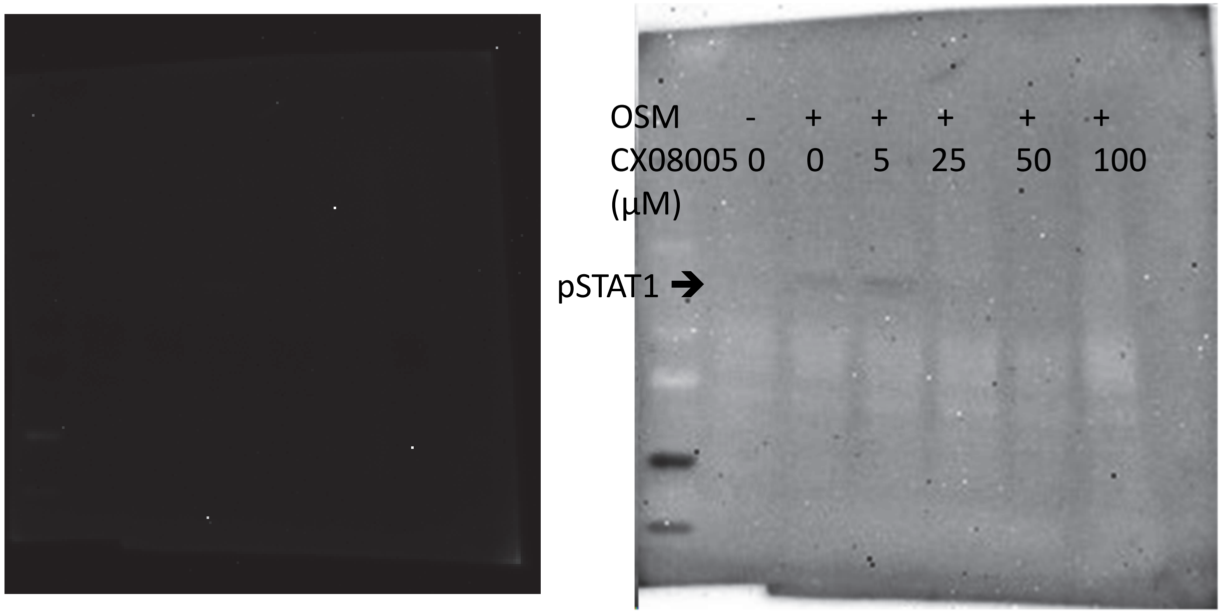

Figure 4A

Replicate 2

OSM	+	+	+	+	+	-
PVSN (µM)	500	90	60	30	0	0

pSTAT1 →

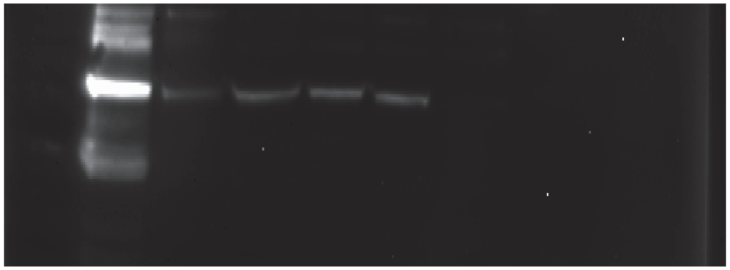

Replicate 3

OSM	+	-	-	-	+	-
PVSN (µM)	500	500	250	125	0	0

pSTAT1 →

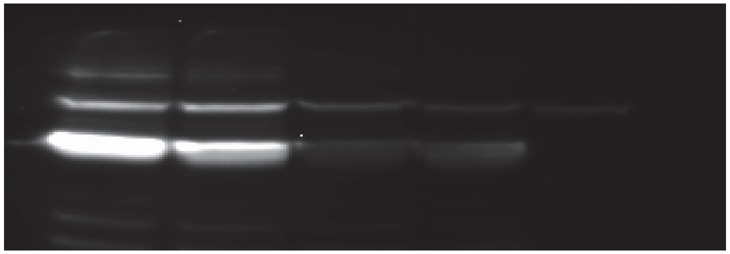

Figure 4B

PVSN and PVS 500 µM

Replicate 2

	PVSN		PVS			
OSM	+	-	+	-	+	-

pSTAT1 →

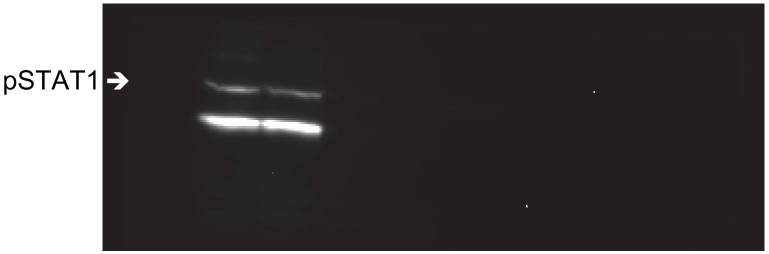

Replicate 3

	PVSN		PVS			
OSM	+	-	+	-	+	-

pSTAT1 →

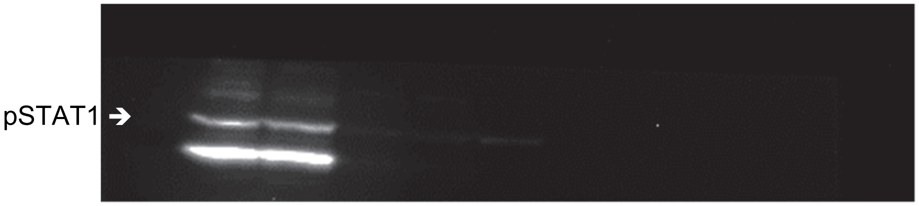

Supplement: Figure S6 — Original images for Figures 2–4. [file peerj-08-9504-s007.pdf]
